# Capivasertib as a Therapeutic Agent for Breast Cancer: Targeting AKT to Overcome Endocrine Resistance

**DOI:** 10.3390/jcm15103803

**Published:** 2026-05-15

**Authors:** Christos Damaskos, Nikolaos Garmpis, Nikolaos Arkadopoulos, Nikolaos V. Michalopoulos, Anna Garmpi, Miltiadis-Panagiotis Papandroudis, Eleni I. Effraimidou

**Affiliations:** 1 Department of Emergency Surgery, Laiko General Hospital, 11527 Athens, Greece; 2 N.S. Christeas Laboratory of Experimental Surgery and Surgical Research, Medical School, National and Kapodistrian University of Athens, 11527 Athens, Greece; 3 Department of Colorectal Surgery, Guys and St Thomas’ Hospital, London SE1 7EH, UK; 4 4th Department of Surgery, Attikon University Hospital, Medical School, National and Kapodistrian University of Athens, Chaidari, 12462 Athens, Greece; narkadopoulos@med.uoa.gr; 5 First Department of Propedeutic Surgery, Hippocration General Hospital, Medical School, National and Kapodistrian University of Athens, 11527 Athens, Greece; nmichal@med.uoa.gr; 6 8th Department of Internal Medicine, Ygeia General Hospital, 15123 Athens, Greece; annagar@windowslive.com; 7 Medical School, University of Plovdiv, 4002 Plovdiv, Bulgaria; manchmilt@gmail.com; 8 Breast Unit, First Surgical Department, University Hospital of Alexandroupolis, Democritus University of Thrace, Dragana, 68100 Alexandroupolis, Greece; eeffraem@med.duth.gr

**Keywords:** capivasertib, AKT inhibitors, breast cancer, breast tumors, endocrine resistance

## Abstract

**Background/Objectives**: Capivasertib is a selective pan-AKT inhibitor recently approved in combination with fulvestrant for the treatment of hormone receptor-positive (HR+)/HER2- breast cancer with alterations in the PI3K/AKT pathway. The PI3K/AKT/mTOR signaling cascade represents a critical indication of endocrine resistance and tumor progression in this subtype of breast cancer. The present review summarizes current clinical data regarding the efficacy of capivasertib, either as monotherapy or in combination with other therapeutic agents and discusses emerging biomarkers and mechanisms of resistance. **Methods**: A literature search of the PubMed database was conducted to identify clinical trials evaluating capivasertib in breast cancer. Studies on capivasertib as monotherapy or in combination with fulvestrant, paclitaxel, or olaparib were included. **Results**: Findings from phase I–III clinical trials indicate that capivasertib in combination with fulvestrant significantly prolongs progression-free survival in patients with HR+/HER2- advanced breast cancer, particularly in tumors containing PIK3CA, AKT1, or PTEN alterations. Drug combination approaches with paclitaxel or olaparib have demonstrated additive or synergistic effects in triple-negative and DNA repair-deficient contexts, respectively. Monotherapy studies confirm effective pathway inhibition with modest clinical benefit, primarily in AKT1-mutant tumors. Translational analyses suggest that persistent mTORC1-mediated protein synthesis and compensatory signaling activation contribute to acquired resistance. **Conclusions**: Capivasertib constitutes a clinically validated therapeutic approach for the inhibition of AKT signaling in breast cancer. Its efficacy is most evident when combined with endocrine therapy; however, optimization of patient selection and rational combination strategies remains necessary to overcome resistance associated with mTORC1 activation and signaling redundancy.

## 1. Introduction

Breast cancer remains the most frequently diagnosed malignancy and a leading cause of cancer-related mortality among women worldwide [1]. Approximately 70% of all breast cancer cases are hormone receptor-positive and human epidermal growth factor receptor 2-negative (HR+/HER2-), a subtype primarily managed with endocrine therapy (ET) [2,3]. ET, including aromatase inhibitors, selective estrogen receptor modulators (SERMs), and selective estrogen receptor degraders (SERDs) such as fulvestrant, represents the most significant treatment approach for HR+/HER2- advanced breast cancer, including cases with visceral involvement, except in the setting of visceral crisis [4,5].

Although ET provides substantial clinical benefit, the majority of patients eventually develop endocrine resistance, resulting in disease progression [4,6]. During the past decade, several targeting therapeutic agents have been introduced to overcome or delay endocrine resistance, most notably inhibitors of cyclin-dependent kinases 4 and 6 (CDK4/6) and components of the phosphatidylinositol 3-kinase (PI3K)/AKT/mammalian target of rapamycin (mTOR) pathway [7,8]. CDK4/6 inhibitors such as palbociclib, ribociclib, and abemaciclib in combination with ET have become the current standard first-line therapy for HR+/HER2- advanced breast cancer, demonstrating significant improvements in progression-free and overall survival [9,10]. However, the development of resistance to these therapeutic agents results in inefficient CDK4/6 inhibition, leaving the therapeutic options restricted [11].

Aberrant activation of the PI3K/AKT/mTOR signaling pathway is one of the most common mechanistic pathways underlying endocrine resistance, occurring in up to 40% of HR+/HER2- breast cancers [12,13]. The introduction of PI3K inhibitors such as alpelisib has improved clinical outcomes in tumors with PIK3CA mutations, yet their efficacy is restricted to this subgroup and is often limited by considerable toxicity [14,15]. Consequently, therapeutic efforts have been shifted toward the downstream of AKT kinase, which represents a critical regulatory kinase with broader therapeutic potential. AKT kinase plays a pivotal role in cellular survival, growth, and metabolic regulation, and its dysregulation has been implicated in resistance to both endocrine and targeted therapies [16,17].

**Figure 1:**
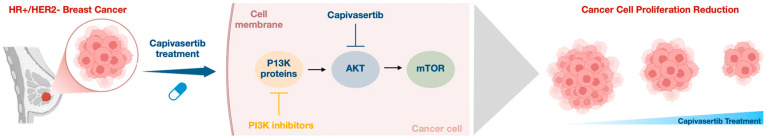
Capivasertib (AZD5363) in HR+/HER2- breast cancer. Capivasertib is a selective pan-AKT inhibitor that targets this central signaling node downstream of AKT, thereby reducing cellular proliferation and survival and potentially restoring sensitivity to endocrine therapy in resistant tumors.

Capivasertib (AZD5363) is a potent, selective, oral pan-AKT inhibitor designed to target this key signaling node [18]. By inhibiting all three AKT isoforms, capivasertib effectively suppresses downstream pathway activation, restores sensitivity to endocrine therapy, and induces antiproliferative effects across multiple breast cancer models (Figure 1).

Up to now, clinical studies have shed light on the therapeutic potential of capivasertib administered either as monotherapy or in combination with other therapeutic agents, particularly in endocrine-resistant and PIK3CA-mutant cases.

Given these advances, a comprehensive understanding of the currently available clinical data supporting the use of capivasertib in breast cancer is essential. The present review summarizes the rationale for AKT inhibition in breast cancer and discusses its emerging therapeutic potential within the current treatment landscape, highlighting remaining challenges and directions for future research.

## 2. Materials and Methods

A structured literature search was conducted in PubMed to identify peer-reviewed English-language articles reporting clinical and translational data on capivasertib in breast cancer. The search used combinations of the following terms: “capivasertib”, “AZD5363”, “AKT inhibitor”, “breast cancer”, “HR-positive breast cancer”, “HER2-negative breast cancer”, “triple-negative breast cancer”, “endocrine resistance”, “fulvestrant”, “paclitaxel”, and “olaparib”. Reference lists of relevant articles were also screened to identify additional studies. Priority was given to phase I-III clinical trials, randomized controlled trials, subgroup analyses, safety analyses, quality-of-life analyses, and translational studies directly relevant to capivasertib activity, biomarkers, and resistance mechanisms in breast cancer. Because the search was limited to PubMed and was not designed as a formal systematic review across multiple databases, the present article should be interpreted as a narrative review with a structured search strategy.

This narrative review summarizes the current clinical evidence supporting capivasertib in breast cancer, with emphasis on endocrine resistance, biomarker-selected populations, combination strategies, and emerging resistance mechanisms.

## 3. Results

### 3.1. Capivasertib Administration

Capivasertib has been administered either as monotherapy or in combination with other chemotherapeutic agents. The use of capivasertib in combination regimens has been guided by both biological rationale and clinical context. In HR+/HER2- breast cancer, capivasertib was combined with fulvestrant to overcome endocrine resistance, particularly in tumors with PIK3CA, AKT1, or PTEN alterations, where PI3K/AKT pathway activation is implicated in resistance to endocrine therapy. In triple-negative breast cancer, the combination with paclitaxel was investigated because this subtype frequently exhibits PI3K/AKT pathway dysregulation and preclinical studies suggested synergistic effects between AKT inhibition and taxane-based chemotherapy [19]. The combination of capivasertib with olaparib was explored in tumors with features of DNA repair deficiency, based on evidence that AKT inhibition can impair homologous recombination and thereby enhance sensitivity to PARP inhibition [20]. Together, these combination strategies reflect an effort to match AKT inhibition with the dominant resistance mechanisms or therapeutic vulnerabilities present in distinct breast cancer subtypes.

#### 3.1.1. Capivasertib as Monotherapy

In exploring the clinical activity and safety of capivasertib (AZD5363) as a single therapeutic agent in patients with advanced solid tumors, including breast cancer, several studies demonstrated that capivasertib is a potent, selective pan-AKT inhibitor with activity against AKT1, AKT2, and AKT3 isoforms, capable of suppressing downstream signaling of the PI3K/AKT/mTOR pathway, a key driver of tumor growth and endocrine resistance.

Phase I dose-escalation and expansion studies evaluated oral capivasertib using continuous and intermittent dosing schedules in heavily pretreated patients with advanced solid tumors. The recommended phase II dose (RP2D) was defined as 480 mg twice daily, on a 4-days-on/3-days-off schedule, based on safety, pharmacokinetics (PK), and pharmacodynamic data. Treatment-related adverse events were mainly gastrointestinal (diarrhea, nausea, vomiting), dermatological (rash), and metabolic (hyperglycemia), consistent with on-target AKT inhibition. Most events were grade 1–2 in severity and were resolved with dose interruption or reduction. Pharmacodynamic studies confirmed robust inhibition of AKT signaling, as evidenced by decreased phosphorylation of downstream AKT substrates (e.g., GSK3β and PRAS40) in peripheral blood mononuclear cells and tumor biopsies (Table 1, Entries 1, 2) [21,22].

In a first-in-human, multicenter, phase I dose-escalation and expansion study, patients with advanced solid malignancies, including breast, endometrial, and prostate cancer, received oral capivasertib at doses ranging from 80 to 800 mg twice daily on continuous or intermittent schedules (4-days-on/3-days-off or 2-days-on/5-days-off). The recommended phase II dose was established at 480 mg twice daily on the intermittent 4-days-on/3-days-off schedule. Capivasertib demonstrated dose-dependent target inhibition and antitumor activity, particularly in tumors containing PIK3CA, AKT1, or PTEN alterations. Partial responses were observed predominantly in patients with AKT1 E17K-mutant breast cancer, while disease stabilization occurred across tumor types. The most frequent adverse events included diarrhea, rash, nausea, fatigue, and hyperglycemia, consistent with AKT pathway inhibition, and were generally manageable through dose adjustments (Table 1, Entry 3) [23].

In a complementary analysis, Voronova et al. evaluated the cardiovascular safety profile of capivasertib in 180 patients with advanced solid tumors using concentration-QT modeling. Across doses up to 800 mg, the predicted mean Fridericia-corrected QT interval (ΔQTcF) change at steady-state exposure for the therapeutic dose (400 mg twice daily) was 3.97 ms, with an upper 90% confidence limit of 5.07 ms—well below the 10 ms threshold of clinical concern. No patients experienced QTcF > 480 ms or ΔQTcF > 60 ms, confirming the absence of clinically significant pro-arrhythmic effects. Collectively, phase I studies established the safety, tolerability, and pharmacodynamic efficacy of capivasertib as a monotherapy, defining the intermittent dosing as optimal and confirming activity in PIK3CA/AKT1/PTEN-altered tumors (Table 1, Entry 4) [24].

Overall, evidence from phase I and expansion studies indicates that capivasertib as a monotherapy demonstrates manageable toxicity, effective target inhibition, and modest antitumor activity, particularly in AKT1-mutant ER-positive breast cancer.

#### 3.1.2. Capivasertib in Combination with Fulvestrant

The initial clinical evidence supporting the therapeutic potential of capivasertib in combination with endocrine therapy was derived from early-phase expansion studies evaluating AKT inhibition in estrogen receptor-positive (ER+) breast cancer.

In a phase I expansion trial, Smyth et al. assessed capivasertib (AZD5363) administered as monotherapy or in combination with fulvestrant in patients with metastatic ER+ breast cancer containing AKT1E17K mutations. Capivasertib was administered at a dose of 400 mg twice daily on an intermittent 4-days-on/3-days-off schedule, together with standard-dose fulvestrant (500 mg intramuscularly every 28 days, with an additional loading dose on day 15 of the first cycle). The combination was well tolerated and demonstrated encouraging antitumor activity, achieving objective response rates of approximately 36% in fulvestrant-pretreated and 20% in fulvestrant non-pretreated patients. These findings provided early clinical findings supporting the role of AKT inhibition in endocrine-resistant disease and justified subsequent phase II and III investigations (Table 1, Entry 5) [25].

Building upon these preliminary observations, the phase II FAKTION trial provided the first randomized findings supporting the clinical benefit of capivasertib in combination with fulvestrant in postmenopausal women with HR+/HER2- advanced breast cancer who had relapsed or progressed during or after aromatase inhibitor (AI) therapy. In this double-blind, placebo-controlled study, patients received fulvestrant 500 mg (days 1 and 15 of cycle 1, then every 28 days) in combination with either capivasertib 400 mg twice daily (4-days-on/3-days-off) or placebo, starting on day 15 of the first cycle. This therapeutic agent combination significantly improved median progression-free survival (PFS) to 10.3 months compared to 4.8 months in the control group (hazard ratio [HR] 0.58, 95% CI 0.39–0.84; *p* = 0.0044). The treatment was generally well tolerated, with the most frequent adverse events being diarrhea, rash, and hypertension (Table 1, Entry 6) [26]. An updated analysis further reported durable clinical benefit, as no statistically significant differences in PFS or overall survival (OS) in the treated with capivasertib group versus the placebo group were observed, demonstrating AKT inhibition as a viable strategy for overcoming endocrine resistance (Table 1, Entry 7) [27].

Subsequent data from the phase III CAPItello-291 trial established capivasertib in combination with fulvestrant as an effective therapeutic option for patients with HR+/HER2- advanced breast cancer. This study enrolled 708 patients who had progressed on or after AI therapy, with or without prior exposure to a CDK4/6 inhibitor. Participants were randomized to receive capivasertib 400 mg twice daily (4-days-on/3-days-off) plus fulvestrant 500 mg intramuscularly (every 14 days for the first three injections, then every 28 days), or matching placebo plus fulvestrant. The trial demonstrated a significant improvement in progression-free survival, both in the overall population and in tumors containing PIK3CA, AKT1, or PTEN alterations, confirming the therapeutic efficiency of AKT pathway blockade beyond the PIK3CA-mutant disease (Table 1, Entry 8) [28].

Further analysis of data from the phase III CAPItello-291 trial refined the safety and tolerability profile of the capivasertib plus fulvestrant treatment approach. Rugo et al. reported that the most common treatment-related adverse events with grade ≥ 3 were primarily limited to rash (12%), diarrhea (9%), and hyperglycemia (2%). Permanent discontinuation occurred in approximately 13% of patients, whereas dose interruptions or reductions (to 320 mg or 200 mg) were effective in managing toxicity without compromising efficacy (Table 1, Entry 9) [29].

Quality-of-life outcomes from the phase III CAPItello-291 trial, presented by Oliveira et al., indicated that patient-reported global health status and functionality remained stable throughout treatment, suggesting that the addition of capivasertib did not result in meaningful deterioration of patient well-being (Table 1, Entry 10) [30]. Regional subgroup analysis from Japan (Table 1, Entry 11) and China/Taiwan (Table 1, Entry 12) confirmed the consistency of efficacy and safety outcomes of this therapeutic approach across ethnic groups and healthcare settings, supporting the external validity of CAPItello-291 results [31,32].

In summary, these studies provide robust clinical evidence that AKT inhibition with capivasertib in combination with fulvestrant offers a clinically meaningful benefit in patients with HR+/HER2- advanced breast cancer following endocrine therapy. The consistent improvement in progression-free survival across populations, together with manageable toxicity and preserved quality of life, highlights this therapeutic agent combination as a key option for patients progressing after CDK4/6 inhibitor-based regimens.

#### 3.1.3. Capivasertib in Combination with Paclitaxel

Preclinical studies suggested a potential synergistic effect between AKT inhibition and taxane-based chemotherapy, providing the rationale for combining capivasertib with paclitaxel in triple-negative breast cancer (TNBC), a subtype characterized by high PI3K/AKT/mTOR pathway activation and limited therapeutic options.

The first clinical results were provided by Turner et al. in a phase I dose-escalation and expansion study evaluating capivasertib in combination with paclitaxel as first-line therapy in patients with metastatic TNBC, determining the safety and tolerability of the RP2D of capivasertib plus paclitaxel. Patients received weekly paclitaxel at 90 mg/m^2^ on days 1, 8, and 15 of each 28-day cycle, combined with oral capivasertib administered intermittently (400 mg twice daily, 4-days-on/3-days-off). The combination was generally well tolerated, with manageable gastrointestinal and dermatologic adverse events consistent with AKT inhibition. Pharmacodynamic analysis confirmed target inhibition, and preliminary antitumor activity was observed, particularly in tumors containing PIK3CA/AKT1/PTEN alterations (Table 1, Entry 13) [33]. These findings established the RP2D and supported the design of subsequent randomized trials.

Building upon these results, the phase II PAKT trial provided the first randomized evidence of clinical benefit from the addition of capivasertib to paclitaxel in previously untreated metastatic TNBC. A total of 140 women were randomized in a 1:1 ratio to receive either paclitaxel (90 mg/m^2^ on days 1, 8, and 15 of each 28-day cycle) plus capivasertib 400 mg twice daily (days 2–5, 9–12, and 16–19) or placebo. The primary endpoint was progression-free survival, with secondary endpoints included OS, objective response rate (ORR), and safety. In the overall population, median PFS was 5.9 months in the capivasertib group versus 4.2 months in the placebo group (HR 0.74, 95% CI 0.50–1.08; one-sided *p* = 0.06), while median OS was significantly prolonged (19.1 vs. 12.6 months; HR 0.61, 95% CI 0.37–0.99; *p* = 0.04). In the PIK3CA/AKT1/PTEN-altered subgroup, the benefit was more pronounced, with median PFS of 9.3 months versus 3.7 months (HR 0.30, 95% CI 0.11–0.79; *p* = 0.01). The most frequent grade ≥ 3 adverse events were diarrhea (13%), rash (4%), fatigue (4%), and infection (4%), all of which were manageable with dose modifications. The study demonstrated that capivasertib could enhance paclitaxel efficacy, particularly in molecularly defined TNBC subsets exhibiting PI3K/AKT pathway activation (Table 1, Entry 14) [34].

More recently, Zhang et al. conducted a multicenter, open-label phase I trial further evaluating capivasertib combined with paclitaxel in advanced TNBC. Patients received weekly paclitaxel (80 mg/m^2^) together with oral capivasertib 400 mg twice daily on the 4-days-on/3-days-off doses. The study confirmed the feasibility of the treatment approach and reported consistent improvements in PFS and OS, particularly in PIK3CA/AKT1/PTEN-altered tumors, highlighting the predictive value of pathway activation for treatment benefit. Safety outcomes were in accordance with previous studies, with diarrhea and rash being the most common adverse events and a low incidence of treatment discontinuation due to toxicity (Table 1, Entry 15) [35].

Overall, clinical data support that the addition of capivasertib to paclitaxel provides a modest but clinically meaningful survival benefit in TNBC, especially among patients with PI3K/AKT pathway alterations. The favorable safety profile and durable responses observed in biomarker-selected populations highlight the potential of AKT inhibition as a rational strategy to improve outcomes in this aggressive breast cancer subtype.

#### 3.1.4. Capivasertib in Combination with Olaparib

Preclinical data indicate that PI3K/AKT pathway inhibition suppresses homologous recombination repair genes such as BRCA1/2, making tumors sensitive to PARP inhibitors, while PARP blockade (PARP inhibitors to block the PARP enzyme, which is involved in DNA repair) induces AKT activation, repairing. This dual inhibition was therefore proposed as a synergistic strategy to overcome DNA repair–mediated resistance.

Westin et al. evaluated this approach in a phase Ib study, combining olaparib 300 mg twice daily with capivasertib 400 mg twice daily (4-days-on/3-days-off) in patients with recurrent endometrial, triple-negative breast, or ovarian cancer. Among 38 heavily pretreated patients, the therapeutic approach was tolerable, with nausea, anemia, and diarrhea to be the most common adverse events. The objective response rate was 19%, with a clinical benefit rate of 41%, including activity in BRCA-wild-type tumors. Correlative analysis showed that treatment response was associated with effective AKT blockade, enhanced DNA damage signaling, and immune activation, whereas resistance correlated with persistent mTOR and RAS-MAPK pathway activity. Patient-derived xenograft models of PARP-resistant TNBC confirmed synergistic tumor inhibition with the olaparib-capivasertib combination (Table 1, Entry 16) [36].

Overall, dual AKT and PARP inhibition demonstrated biological synergy, manageable toxicity, and clinical activity across solid tumors, supporting further investigation in homologous PARP-resistant diseases.

Table 1 summarizes the aforementioned studies evaluating the capivasertib in breast cancer.

### 3.2. Biomarkers and Resistance Mechanisms

The preclinical evaluation of capivasertib by Davies et al. established its selectivity and mechanistic basis for targeting the AKT pathway across diverse tumor models. Capivasertib exhibited strong inhibition of all three AKT isoforms and effectively suppressed phosphorylation of key downstream substrates, including PRAS40, GSK3β, and S6. In vitro, capivasertib reduced proliferation in cell lines containing PIK3CA, AKT1, or PTEN alterations, while in vivo studies demonstrated significant tumor growth inhibition and pathway suppression in xenograft models (Table 2, Entry 1) [37]. These findings validated AKT inhibition as a therapeutic strategy and provided the pharmacologic rationale for subsequent clinical trials in breast cancer. In CAPItello-291, pathway alteration status was determined centrally using next-generation sequencing of tumor tissue with the FoundationOne CDx assay. The biomarker-altered population included patients whose tumors harbored qualifying alterations in PIK3CA, AKT1, or PTEN. These comprised activating alterations in PIK3CA or AKT1, and inactivating or loss-of-function alterations in PTEN. Current regulatory labeling similarly recommends selecting patients for capivasertib plus fulvestrant based on the presence of one or more PIK3CA/AKT1/PTEN alterations detected using an approved diagnostic test.

Pharmacodynamic confirmation of target engagement in humans was provided by the STAKT presurgical study in which capivasertib administration to ER-positive breast cancer patients prior to surgery resulted in marked reductions in phosphorylated PRAS40, GSK3β, and S6, together with reduced Ki67 proliferation indices (Table 2, Entry 2) [38]. These findings demonstrated effective pathway suppression at clinically achievable doses, supporting translational consistency between preclinical and clinical settings.

More recently, Sobsey et al. provided proteomic data clarifying resistance mechanisms within PIK3CA-altered tumors treated with capivasertib as a monotherapy. Tumors achieving durable disease control (≥12 weeks) exhibited reduced expression of mTORC1-regulated translational proteins compared with non-responders, while persistent mTORC1 activation correlated to intrinsic resistance despite AKT inhibition. These observations were further validated in PIK3CA- and AKT1-mutant breast cancer cell lines, indicating that increased mTORC1-driven translational output may act as a compensatory mechanism limiting response to therapy.

These preclinical and translational studies confirm that while capivasertib achieves potent inhibition of AKT signaling and antiproliferative activity, sustained mTORC1 activation remains a principal determinant of resistance (Table 2, Entry 2) [39].

Table 2 summarizes the aforementioned findings relative to the capivesartib activity and resistance in breast cancer.

Such insights highlight the importance of future efforts to refine patient selection and develop rational combination strategies that extend the clinical benefit of AKT inhibition in breast cancer.

## 4. Discussion

The clinical development of capivasertib has established a proof-of-concept for AKT inhibition as a therapeutic approach in breast cancer. Across multiple studies, capivasertib demonstrated consistent pathway suppression, a predictable safety profile, and measurable clinical benefit in both HR+/HER2- and TNBC. The greatest efficacy was observed in combination with fulvestrant, where clinically meaningful improvements in median progression-free survival were observed. In CAPItello-291, median progression-free survival improved from 3.6 months with placebo plus fulvestrant to 7.2 months with capivasertib plus fulvestrant in the overall population, and from 3.1 to 7.3 months in patients with PIK3CA/AKT1/PTEN-altered tumors [28]. Similarly, in the phase II FAKTION trial, median progression-free survival improved from 4.8 months with placebo plus fulvestrant to 10.3 months with capivasertib plus fulvestrant [26]. Importantly, the therapeutic benefit extended to both biomarker-positive and biomarker-negative tumors, indicating that aberrant AKT signaling contributes to endocrine resistance. This broad activity supports the role of AKT as a key regulatory node integrating survival and growth signals downstream of multiple receptor pathways.

In the contemporary post-CDK4/6 inhibitor setting, capivasertib plus fulvestrant should be considered within an increasingly biomarker-driven treatment landscape. For patients with tumors harboring PIK3CA/AKT1/PTEN alterations, capivasertib plus fulvestrant represents an endocrine-based targeted option supported by CAPItello-291 [28]. Its clinical positioning overlaps partly with alpelisib plus fulvestrant in PIK3CA-mutated disease, although alpelisib is restricted to PIK3CA-mutant tumors and is frequently limited by metabolic toxicity, particularly hyperglycemia. In contrast, capivasertib targets a broader PI3K/AKT pathway-altered population, including AKT1 and PTEN alterations [5,15,29]. For patients with ESR1-mutated tumors and preserved endocrine sensitivity, oral SERDs such as elacestrant represent another endocrine-based option, especially when avoidance of combination targeted toxicity is preferred [40,41]. In patients with rapidly progressive disease, endocrine-refractory disease, high visceral burden, or HER2-low/HER2-ultralow status, antibody-drug conjugates such as Trastuzumab may become more relevant, usually after exhaustion of appropriate endocrine-based strategies [41]. Because direct comparative sequencing trials are lacking, treatment selection should integrate molecular profiling, prior exposure to CDK4/6 and PI3K-pathway inhibitors, endocrine sensitivity, comorbidities, toxicity profile, disease tempo, and patient preference.

Despite these advances, the overall magnitude of benefit remains modest, and responses are often temporary. In the CAPItello-291 trial, the median improvement in progression-free survival was approximately 3–4 months, suggesting that capivasertib monotherapy or dual blockade with endocrine agents may be insufficient to achieve durable disease control. Mechanistically, limited efficacy has been linked to pathway repetition within the PI3K/AKT/mTOR network and repairing activation of parallel signaling routes, particularly RAS/MAPK and mTORC1-mediated translation. Preclinical and proteomic analyses have shown that persistent mTORC1 activity and increased translational output represent major resistance mechanisms, explaining why some genetically defined tumors do not derive sustained benefit from AKT inhibition. Moreover, pharmacodynamic studies indicate that pathway reactivation occurs rapidly after drug withdrawal, highlighting the need to optimize dosing schedules and exposure dynamics.

Tolerability remains an important clinical consideration. Capivasertib exhibits a well-characterized and manageable safety profile, with gastrointestinal adverse events (notably diarrhea) and dermatologic reactions (rash) being the most frequent causes of temporary dose interruption. Hyperglycemia, typically low grade, reflects on-target AKT inhibition and requires proactive monitoring. The intermittent 4-days-on/3-days-off dosing schedule was designed to balance efficacy and toxicity; however, the long-term metabolic consequences of sustained AKT inhibition remain under investigation. Effective clinical use will depend on clinician familiarity with early recognition and management of these adverse events to maintain adherence and ensure optimal therapeutic exposure.

Practical toxicity management is central to maintaining treatment adherence with capivasertib [29]. Patients should be educated to report diarrhea early, and management should include prompt oral hydration, antidiarrheal therapy, and temporary treatment interruption or dose reduction for persistent or higher-grade symptoms. Cutaneous adverse events, including maculopapular rash and pruritus, require early recognition, supportive skin care, antihistamines, topical corticosteroids, and dermatology input for severe or recurrent reactions [29,42]. Hyperglycemia should be anticipated as an on-target effect of AKT inhibition; fasting glucose and HbA1c should be assessed before treatment and monitored during therapy, particularly in patients with diabetes, prediabetes, obesity, or concomitant corticosteroid exposure [42]. Antihyperglycemic treatment may be required, and capivasertib should be interrupted, dose-reduced, or discontinued according to severity and persistence of metabolic toxicity [29].

This review has several limitations. First, it is a narrative review supported by a structured literature search and should not be interpreted as a formal systematic review or meta-analysis. Although PubMed was searched and an additional confirmatory search of the Cochrane Library/CENTRAL was performed, relevant unpublished studies, conference-only abstracts, or ongoing trials may not have been fully captured. Second, the included evidence is heterogeneous, comprising phase I dose-escalation studies, randomized phase II and III trials, subgroup analyses, safety analyses, quality-of-life analyses, and translational studies. These studies differ in patient population, molecular selection, treatment regimen, dosing schedule, and reported endpoints, limiting direct cross-trial comparison. Third, meta-analysis was not performed because of this heterogeneity and the limited number of comparable breast cancer-specific monotherapy cohorts. Finally, direct sequencing data comparing capivasertib plus fulvestrant with other post-CDK4/6 options, such as alpelisib, elacestrant, trastuzumab deruxtecan, or sacituzumab govitecan, remain limited. Therefore, treatment positioning should be interpreted in the context of available trial data, biomarker profile, prior therapy, toxicity considerations, and clinical judgment.

## 5. Conclusions

Capivasertib represents the first clinically validated pan-AKT inhibitor to receive regulatory approval, in combination with fulvestrant, for the treatment of HR+/HER2- breast cancer with PI3K pathway alterations. Data from phase I-III studies consistently demonstrate that AKT inhibition can overcome endocrine resistance and prolong progression-free survival in both biomarker-selected and unselected populations. When combined with taxane-based chemotherapy or PARP inhibitors, capivasertib also exhibits additive or synergistic efficacy, extending its therapeutic potential beyond hormone receptor-positive disease. Collectively, these findings exhibit the PI3K/AKT/mTOR cascade as a critical therapeutic area in breast cancer and demonstrate capivasertib as a promising therapeutic agent.

The identification of persistent mTORC1-driven translational output as a potential resistance mechanism provides a rationale for therapeutic strategies that co-target multiple nodes of the PI3K/AKT/mTOR axis. Although direct clinical evidence for combined AKT and mTOR inhibition in breast cancer remains limited, early-phase studies have explored related approaches, including capivasertib-containing combinations and mTORC1/2 inhibitor-based regimens. Future trials should determine whether rational co-targeting of AKT, mTORC1/2, or parallel compensatory pathways can overcome adaptive resistance while maintaining an acceptable toxicity profile.

## Figures and Tables

**Table 1:** Summary of key clinical and outcomes of capivasertib in breast cancer.

Entry	Study	Study/Population	Drug Dosage	Results
1	Banerji et al. 2018 [21]	Phase I, dose escalating study (NCT01226316)/90	Capivasertib 320 mg (continuous), 480 mg (4/7) and 640 mg (2/7)	Optimal dose: 480 mg. Adverse events: diarrhea, nausea and hyperglycemia
2	Tamura et al. 2016 [22]	Phase I dose escalating study/41	Capivasertib: 80–400 mg (continuous), 360–480 mg, (4/3) and 640 mg (2/5)	Intermittent dosing was more tolerable than continuous dosing
3	Kalinsky et al. 2022 [23]	NCI-MATCH trial/35	Capivasertib: 480 mg or 320 mg twice daily (4 on/3 off)	ORR 28%, grade 3 adverse events: hyperglycemia and rash, 1 patient had a grade 4 hyperglycemic adverse event
4	Voronova et al. 2022 [24]	Dose expansion study/180	Capivasertib doses ranging from 80 mg to 800 mg twice daily (4 on/3 off), dose expansion at 480 mg	Positive correlation between concentration and ΔQTcF
5	Smyth et al. 2020 [25]	Phase I study (NCT01226316)/43	Capivasertib 400 mg twice daily (4 on/3 off) + fulvestrant 500 mg every 28 days, an additional dose on day 15	ORR of 36% (pretreated), 20% (non-pretreated), grade ≥ 3 adverse events: rash, hyperglycemia & diarrhea
6	Jones et al. 2020 [26]	FRAKTION: Phase II HR+/HER2- placebo-controlled study (NCT01992952)/140	Capivasertib 400 mg twice daily (4 on/3 off) + fulvestrant 500 mg (days 1 and 15 of cycle 1, then every 28 days)	Median PFS 10.3 vs. 4.8 (placebo) (HR 0.58, *p* = 0.0044), diarrhea, rash and hypertension
7	Howell et al. 2022 [27]	FRAKTION (update): Phase II trial, OS, PFS updated (NCT01992952)/140	Capivasertib 400 mg twice daily (4 on/3 off) + fulvestrant 500 mg (days 1 and 15 of cycle 1, then every 28 days)	No statistically significant differences in PFS or OS
8	Turner et al. 2023 [28]	CAPItello-291: Phase III randomized, double-blind trial (NCT04305496)/708	Capivasertib 400 mg twice daily (4 on/3 off) + fulvestrant 500 mg (days 1 and 15 of cycle 1, then every 28 days)	PFS 7.2 vs. 3.6 (placebo) (HR 0.60), PIK3CA/AKT1/PTEN-altered 7.3 vs. 3.1 mo (HR 0.50)
9	Rugo et al. 2024 [29]	CAPItello-291: Safety analysis/705	Capivasertib 400 mg twice daily (4 on/3 off) + fulvestrant 500 mg (days 1 and 15 of cycle 1, then every 28 days)	Grade ≥ 3 adverse events: rash (12%), diarrhea (9%), hyperglycemia (2%)
10	Oliveira et al. 2024 [30]	CAPItello-291: HRQOL analysis/708	Capivasertib 400 mg twice daily (4 on/3 off) + fulvestrant 500 mg (days 1 and 15 of cycle 1, then every 28 days)	Stable QoL scores, delayed time to deterioration of GHS/QOL and maintained dimensions of HRQOL (except symptoms of diarrhea)
11	Tokunaga et al. 2025 [31]	CAPItello-291: Japan subgroup analysis/78	Capivasertib 400 mg twice daily (4 on/3 off) + fulvestrant 500 mg (days 1 and 15 of cycle 1, then every 28 days)	Consistent efficacy/safety with global trial (HR 0.65; 95% CI 0.29–1.39)
12	Hu et al. 2025 [32]	CAPItello-291: China subgroup analysis/134	Capivasertib 400 mg twice daily (4 on/3 off) + fulvestrant 500 mg (days 1 and 15 of cycle 1, then every 28 days)	PFS 6.9 vs. 2.8 (placebo) (HR 0.51, 95% CI 0.34–0.76). Adverse events: diarrhea (60%) and hyperglycemia (58%)
13	Turner et al. 2019 [33]	BEECH: Phase Ib study ER+/HER2- MBC patients/38	Paclitaxel 90 mg/m^2^ (day 1, 8, 15 of 28-day cycle) + capivasertib 400 mg twice daily (4 on/3 off)	PFS 10.9 vs. 8.4 (placebo) (HR 0.80, *p* = 0.308) 95% CI 0.34–0.76). Adverse events: diarrhea, rash and hyperglycemia
14	Schmid et al. 2020 [34]	PAKT: Phase II trial/140	Paclitaxel 90 mg/m^2^ (day 1, 8, 15) + capivasertib 400 mg twice daily (days 2–5, 9–12, 16–19 every 28 days)	PFS 5.9 vs. 4.2 (placebo) (HR 0.74); OS 19.1 vs. 12.6 (placebo) (HR 0.61), greatest benefit in PIK3CA/AKT1/PTEN-altered
15	Zhang et al. 2025 [35]	Phase I open-label study/16	Paclitaxel 80 mg/m^2^ once weekly (3 weeks on/1 week off) + capivasertib 400 mg twice daily (4 on/3 off)	Events of grade 1–2: 25% patients achieved confirmed partial response and 25% stable disease as best objective response.
16	Westin et al. 2021 [36]	Phase I dose expansion analyses/38	Olaparib 300 mg twice daily + capivasertib 400 mg or 320 mg twice daily (4 on/3 off)	ORR 19%, CBR 41%, main adverse events: anemia, diarrhea, nausea

ORR: objective response rate; PFS: progression-free survival; HR: hazard ratio; OS: overall survival; QoL: quality of life; GHS: global health status; CI: confidence interval; CBR: clinical benefit rate.

**Table 2:** Summary of key clinical and outcomes of capivasertib activity and resistance in breast cancer.

Entry	Study	Study Type	Participants	Drug	Results
1	Davies et al. 2015 [37]	Preclinical characterization	N/A	Capivasertib (oral AKT inhibitor)	↓ pPRAS40, ↓ pS6, tumor growth suppression in xenografts
2	Robertson et al. 2020 [38]	STAKT: Presurgical pharmacodynamic study	16	Capivasertib (oral, short-course pre-surgery)	↓ pPRAS40, ↓ pGSK3β, ↓ pS6, ↓ Ki67, on-target activity confirmed
3	Sobsey et al. 2024 [39]	Translational proteomics in PIK3CA-mut tumors	16	Capivasertib monotherapy samples from phase I	mTORC1-driven translation ↑ in non-responders, resistance mechanism identified

N/A: not applicable; ↑: increased/upregulated; ↓: decreased/downregulated.

## Data Availability

No new data were created in this study. The data supporting the findings are derived from previously published studies, which are cited within the article.

## Abbreviations

The following abbreviations are used in this manuscript:

HR+	Hormone receptor-positive
HER2-	Human epidermal growth factor receptor 2-negative
ET	Endocrine therapy
SERM	Selective estrogen receptor modulators
SERD	Selective estrogen receptor degraders
CDK	Cyclin-dependent kinase
PI3K	Phosphatidylinositol 3-kinase
mTOR	Mammalian target of rapamycin
RP2D	Recommended phase II dose
PK	Pharmakokinetics
PFS	Progression-free survival
HR	Hazard ratio
CI	Confidence interval
OS	Overall survival
TNBC	Triple-negative breast cancer
ORR	Objective response rate
PD	Pharmacodynamic
QoL	Quality of life
GHS	Global health status
CBR	Clinical benefit rate
